# Prognostic characteristics and clinical response to immunotherapy targeting programmed cell death 1 for patients with advanced gastric cancer with liver metastases

**DOI:** 10.3389/fimmu.2022.1015549

**Published:** 2022-09-21

**Authors:** Huayuan Liang, Zhiwei Li, Zhicheng Huang, Chaorui Wu, Yaopeng Qiu, Yanrui Liang, Xinhua Chen, Fengping Li, Zhou Xu, Guoxin Li, Hao Liu, Liying Zhao

**Affiliations:** Department of General Surgery, Nanfang Hospital, The First School of Clinical Medicine, Southern Medical University, Guangzhou, China

**Keywords:** liver metastases, gastric cancer, immunotherapy, programmed cell death 1, immune checkpoint inhibitor

## Abstract

**Background:**

The specific efficacy of immunotherapy for patients with liver metastases of gastric cancer is unclear. This study set out to explore the treatment response and related prognostic factors for patients with liver metastases of gastric cancer treated with immunotherapy.

**Patients and methods:**

This retrospective cohort study included 135 patients with unresectable advanced gastric cancer. According to the presence of liver metastases and/or first-line treatment with immunotherapy, patients were divided into the following three groups: I-LM(-) group(patients without liver metastases treated with immunotherapy, n=66), I-LM(+) group(patients with liver metastases treated with immunotherapy, n=36), C-LM(+) group(patients with liver metastases treated with chemotherapy and/or target therapy, n=33). Cox regression analyses were used to identify factors associated with survival in all patients and the three groups, respectively.

**Results:**

For the patients with liver metastases treated with immunotherapy, multivariate analysis showed that only the presence of peritoneal metastases was significantly associated with shorter PFS [hazard ratios (HR), 3.23; 95% CI, 1.12-9.32; P=0.030] and the patients with peritoneal metastases had shorter median PFS than patients without peritoneal metastases(3.1 vs 18.4 months; P=0.004), while the objective response rate was 100% in patients with HER2-positive (2 complete radiographic responses and 2 partial responses; 3 of 4 patients were still ongoing benefits [median follow-up time, 15.3 months ; interquartile range(IQR), 6.3-17.9 months]).

**Conclusions:**

The findings suggest that patients with various types of gastric cancer liver metastases respond differently to immune checkpoint inhibitors, HER2-positive patients may derive clinical benefits from immune checkpoint inhibitors, while the presence of peritoneal metastases is associated with resistance.

## Introduction

Globally, gastric cancer is the leading cause of cancer-related deaths, which is usually diagnosed at a late stage, accompanied with distant metastases and poor survival expectations. From the later to the first line of treatment, immunotherapy has made significant strides in advanced gastric cancer. Large clinical trials have demonstrated significant efficacy of immune checkpoint inhibitors combined with chemotherapy and/or targeted therapy for advanced gastric cancer, including the CheckMate-649 trial, the ORIENT-16 trial, and the KEYNOTE-811 trial (1–3). However, the failures of some clinical trials and the high heterogeneity of gastric cancer suggest that immunotherapy for gastric cancer requires identification for the beneficiaries and demands precision treatment (4, 5).

The liver is one of the most common sites of visceral metastasis for a variety of cancers. Preclinical data demonstrate that tumors with liver metastases can disrupt the tumor immune microenvironment and cause tumor immune escape (6, 7). Some clinical findings of relative clinical resistance to immunotherapy and unfavorable outcomes were noted in patients with liver metastases in non-small cell lung cancer, melanoma, colorectal cancer, and other tumor types (8–11). The liver is the most prevalent target organ for hematogenous metastases of gastric cancer, and a large retrospective study showed that the incidence of liver metastases in patients with stage IV gastric cancer was up to about 40% (12). The CheckMate-649 Chinese subgroup analysis with 2-year follow-up showed that the presence of liver metastases was not associated with poor prognosis in patients with advanced gastric cancer treated with immunotherapy [median overall survival (OS) in liver metastases arm vs non-liver metastases arm:14.2 vs 14.8 months] (13). Similarly, the survival benefit of Nivolumab was observed in the ATTRACTION-2 trial (Nivolumab vs placebo) or the ATTRACTION-4 trial (Nivolumab plus chemotherapy vs placebo plus chemotherapy) regardless of liver metastases of gastric cancer (14, 15). However, the REGONIVO trial (Regorafenib plus Nivolumab in Patients with Advanced Gastric or Colorectal Cancer) reported a response rate of 44% and a progressive free survival (PFS) of 5.6 months in 25 patients with advancer gastric cancer who had disease progression with standard chemotherapy. In that study, patients with liver metastases had a response rate of 41.7% (5 of 12), whereas patients with lung metastases without liver involvement had a response rate of 80% (4 of 5) (16). In addition, some retrospective studies suggested that the presence of liver metastases in gastric cancer was associated with rapid disease progression or a lower response rate compared with other metastases (17, 18). The specific efficacy of immunotherapy for patients with liver metastases of gastric cancer remains controversial.

The National Comprehensive Cancer Network (NCCN) guidelines recommend immune checkpoint inhibitors as first-line therapy for advanced gastric cancer (19), and identifying prognostic characteristics associated with immunotherapy in patients with liver metastases could provide references in decision-making regarding such patients. Here, we performed a single-center, retrospective cohort study to explore the prognostic factors and clinical response in advanced gastric cancer patients with liver metastases who were treated with first-line immunotherapy combination therapy (chemotherapy and/or targeted therapy).

## Methods

### Patient population

**Figure 1:**
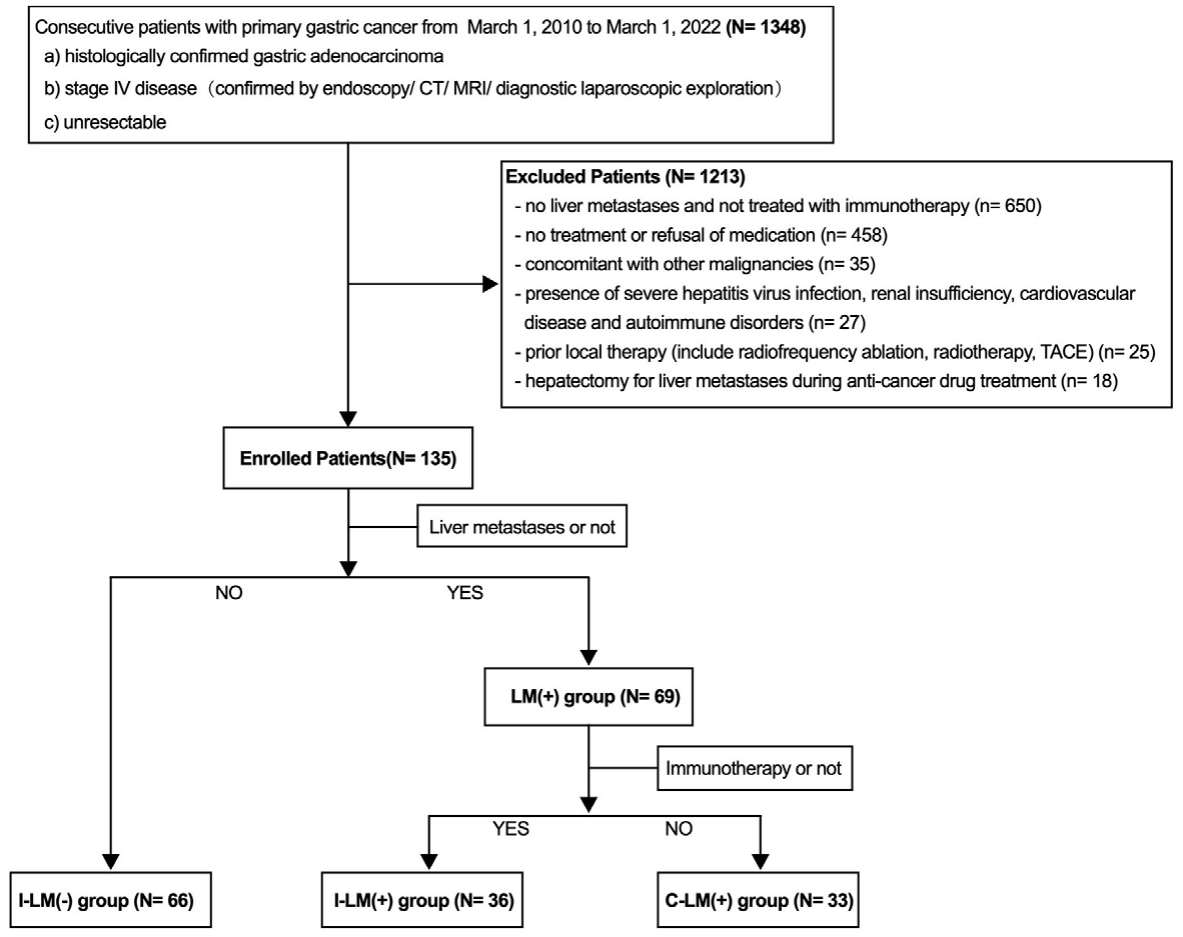
Flowchart of the study population.

This study analyzed clinicopathological data from patients with unresectable advanced gastric cancer who were treated from March 1, 2010 to March 1, 2022, from Nanfang Hospital, Southern Medical University, People’s Republic of China. The flowchart of the study population is shown in **Figure 1**. The exclusion criteria:1) no liver metastases and not treated with immunotherapy, 2) no treatment or refusal of medication, 3) concomitant with other malignancies, 4) presence of severe hepatitis virus infection, renal insufficiency, cardiovascular disease, and autoimmune disorders, 5) prior local therapy (include radiofrequency ablation, radiotherapy, TACE, 6) hepatectomy for liver metastases during anti-cancer drug treatment. Immunotherapy was defined as immune checkpoint inhibitors targeting programmed cell death 1 (PD-1 inhibitor) (details: Toripalimab 70.5%, Sintilimab 16.7%, Nivolumab 6.9%, Pembrolizumab 5.9%). We collected clinicopathologic features including age, gender, Body Mass Index (BMI), primary tumor location and size, distant metastatic organ, eastern cooperative oncology group (ECOG) performance status, and tumor histological grade. Serum alpha-fetoprotein (AFP) and lactate dehydrogenase (LDH) levels were evaluated in patients with liver metastases before the first cycle of immunotherapy. The cut-off value for serum AFP and LDH was 25 ng/mL and 199 ng/mL, which was the upper limit of the normal reference value of AFP and LDH in our hospital laboratory, respectively.

### Investigation of potential biomarkers for immunotherapy or target therapy

All patients treated with PD-1 inhibitors signed informed consent forms, and the previous biopsy samples were sent for further immunohistochemical (IHC) stain, including MLH1, MSH2, MSH6, and PMS2 for mismatch repair protein to confirm the deficient DNA mismatch repair (dMMR) status, HER2 status, Epstein–Barr virus-encoded regions (EBER) status. For patients not treated with immune checkpoint inhibitors, the primary biomarker was HER2 status. HER2 positivity was defined as IHC 3+, or IHC 2+ with HER2 gene amplification by *In Situ* Hybridization (ISH). PD-L1 was assessed by the pharmDx immunohistochemistry assay (PD-L1 IHC 22C3) combined positive score (CPS).

### Clinical response

Individual patients’ imaging scans were evaluated by qualified radiologists during immune checkpoint inhibitors therapy, and tumor responses were judged using the Response Evaluation Criteria in Solid Tumors version 1.1 guidelines. Progression-free survival was defined as the time interval from the date of initiation of treatment to disease progression. Overall survival was defined as the time from antitumor therapy to the date of death from any causes or censorship at the last follow-up. Patients lost to follow-up were censored at the date of the last contact.

### Follow-up

Participants were followed up through outpatient visits and telephone calls. Outpatient examinations included physical examination, laboratory examination (routine blood test, blood biochemical examination, and measurement of AFP, carcinoembryonic antigen (CEA), and carbohydrate antigen 19-9 levels), chest radiography, abdominal ultrasonography, or computed tomography. All patients were followed up once every 3 to 6 months during the first 2 years, once every 6 to 12 months during the following 3 to 5 years, and once every year thereafter. A median follow-up period of 14.5 months was calculated using the reverse Kaplan-Meier method.

### Statistical analysis

The demographic and clinical characteristics of the liver metastases and no liver metastases groups were described using descriptive analysis. The chi-square test or Fisher’s exact test was used for comparisons of categorical variables and the T-test was used for comparisons of continuous variables. Univariate and multivariate Cox regression analyses were used to identify factors associated with PFS, estimating their hazard ratios, and associated 95% confidence intervals, and variables with P <0.05 in the univariable analysis were included in the multivariable model. Median PFS and their associated 95% confidence intervals were calculated by the Kaplan–Meier method. Statistical analyses were performed using SPSS V26 (IBM Corp., Armonk, NY) and GraphPad PRISM (Prism 9.0.2; GraphPad Software, LLC). All statistical tests were 2-sided with a significance threshold of P <0.05.

## Result

### Baseline population characteristics in all patients

A total of 135 patients with stage IV advanced gastric cancer were enrolled in this study, including 66 patients without liver metastases and 36 patients with liver metastases treated with immunotherapy combined with chemotherapy and/or targeted therapy, and 33 patients with liver metastases treated with chemotherapy and/or targeted therapy (**Figure 1**). The demographic information and relevant prognostic variables are as follows (**Supplementary Table 1**). The median age of all the patients was 56 years (IQR, 21-76 years). Males were near twice as many as females and the average BMI was 21.26 (IQR, 11.9-30.0). A total of 116 of 135 patients (85.9%) had an ECOG performance status of 0. The proportions of primary tumors in the upper, middle, and lower parts were 20.0%, 22.2%, and 41.5%, respectively. Nearly half of patients had primary tumors ≥5 cm in size and approximately four-fifths of patients had poorly differentiated histological grades. A portion of patients had distant organ metastases, and the proportions of distant lymph nodes metastases, peritoneal metastases, lung metastases, and bone metastases were 19.3%, 64.4%, 14.1%, and 14.8%, respectively.

All the patients were divided into two groups based on the presence or absence of liver metastases, with 69 patients in the liver metastases group and 66 patients in the non-liver metastasis group. There were no significant differences between groups in age, gender, BMI, ECOG score, histological grade, lung metastases, and bone metastases. However, the proportions of peritoneal metastases and primary tumor size ≥5 cm were significantly higher in the non-liver metastasis group compared with the liver metastases group. Baseline patient demographics are detailed in **Supplementary Table 1** in the Supplement.

### Survival analysis and clinicopathologic characteristics associated with PFS in all patients

**Figure 2:**
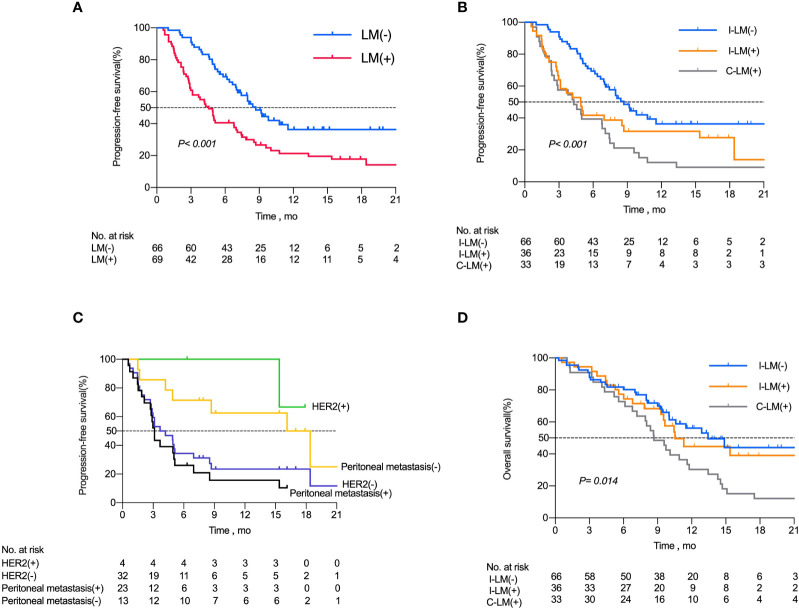
Kaplan-Meier Curves for Progression-Free Survival Among Patients with Advanced Gastric Cancer. **(A)** Kaplan–Meier analysis of progression free survival in all patients with or without liver metastases. **(B)** Kaplan–Meier analysis of progression free survival in I-LM(-) group, I-LM(+) group and C-LM(+) group, respectively. **(C)** Kaplan–Meier analysis of progression free survival in patients with or without peritoneal metastases in I-LM(+) group. **(D)** Kaplan–Meier analysis of overall survival in I-LM(-) group, I-LM(+) group and C-LM(+) group, respectively.

As calculated by the Kaplan–Meier method, the median PFS of the 66 patients without liver metastases was 8.7 months, while that of patients with liver metastases was 4.5 months (P <0.001, **Figure 2A**). The 6-month PFS rates for patients without and with liver metastases were 65.1% (43 of 66) and 40.5% (28 of 69), respectively.

Univariate and multivariate analyses were performed for all patients using Cox regression models. Univariate analysis showed that the patients with peritoneal metastases (HR, 2.19; 95% CI, 1.38-3.47; P=0.001), liver metastases (HR, 2.04; 95% CI, 1.34-3.09; P=0.001), bone metastases (HR, 1.87; 95% CI, 1.11-3.16; P=0.018), distant metastasis sites ≥ 3 (HR, 2.27; 95% CI, 1.49-3.45; P<0.001), and combined immunotherapy (HR, 0.49; 95% CI, 0.32-0.77; P=0.002) were all significantly associated with PFS. A multivariate model including these statistically significant variables maintained that liver metastases (HR, 2.42; 95% CI, 1.42-4.14; P=0.001), peritoneal metastases (HR, 2.07; 95% CI, 1.24-3.46; P=0.005), distant metastasis sites ≥ 3 (HR, 1.69; 95% CI, 1.03-2.78; P=0.038) were the significant factors associated with shorter PFS, and combined immunotherapy (HR, 0.55; 95% Cl, 0.32-0.94; P=0.029) was associated with longer PFS (**Supplementary Table 2**).

### Baseline population characteristics in I-LM(-) group, I-LM(+) group, C-LM(+) group

Based on multivariate analysis with statistically significant variables: liver metastases and combined immunotherapy, all patients were divided into three groups: I-LM(-) group (n=66), I-LM(+) group (n=36), C-LM(+) group (n=33). The three groups were pairwise compared with clinicopathological characteristics(**Table 1**). There were no significant differences between the three groups in terms of gender, age, BMI, histological grade, distant lymph nodes metastases, lung metastases, bone metastases, and biomarkers (CPS, HER2, MMR, EBER). The number of patients with ECOG scores=0 was greater in the I-LM(-) group (90.9%) and the I-LM(+) group (94.4%) than in the C-LM(+) group (66.7%) (all P <0.05). The proportion of patients with primary gastric tumor size ≥ 5 cm was higher in the I-LM(-) group than that in the I-LM(+) group (62.1% vs 38.9%, P=0.024). The proportion of peritoneal metastases was higher in the I-LM(-) group than that in the C-LM(+) group (74.2% vs 45.5%, P=0.005). Additional baseline demographics are detailed in **Table 1**.

**Table 1:** Baseline characteristics of patients before first treatment in the I-LM(-) group, I-LM(+) group, and C-LM(+) group, respectively.

	Patients, No. (%)					
Characteristic	I-LM (-) (n=66)	I-LM (+) (n=36)	C-LM (+) (n=33)	P value^1^	P value^2^	P value^3^
Demographic						
Gender				0.301	0.102	0.558
Male	39 (59.1)	25 (69.4)	25 (75.8)			
Female	27 (40.9)	11 (30.6)	8 (24.2)			
Age, y				0.598	0.769	0.848
<60	42 (63.6)	21 (58.3)	20 (60.6)			
≥60	24 (36.4)	15 (41.7)	13 (39.4)			
Clinical						
BMI,mean(SD)	21.01 (3.37)	22.27 (3.80)	20.65 (3,61)	0.089	0.635	0.061
ECOG				0.803	0.003	0.003
0	60 (90.9)	34 (94.4)	22 (66.7)			
≥1	6 (9.1)	2(5.6)	11 (33.3)			
Tumor location				0.245	0.004	0.507
Upper	14 (22.1)	7 (19.5)	6(18.2)			
Middle	18 (27.3)	8 (22.2)	4 (12.1)			
Lower	30 (45.5)	14(38.9)	12 (36.4)			
Mixed	4 (6.1)	7 (19.4)	11 (33.3)			
Primary tumor size				0.024	0.115	0.581
<5cm	25 (37.9)	22(61.1)	18 (54.5)			
≥5cm	41 (62.1)	14 (38.9)	15(45.5)			
Histological grade				0.187	0.059	0.233
Well or moderately	10 (15.2)	3(8.3)	7 (21.2)			
Poorly differentiated	54 (81.8)	29 (80.6)	21 (63.6)			
Unknown	2(3.0)	4 (11.1)	5 (15.2)			
Site of distant metastases						
Lymph nodes				0.953	0.097	0.137
Yes	15 (22.7)	8 (22.2)	3 (9.1)			
No	51(77.3)	28 (77.8)	30 (90.9)			
Peritoneum				0.273	0.005	0.124
Yes	49(74.2)	23(63.9)	15 (45.5)			
No	17 (25.8)	13 (36.1)	18(54.5)			
Lung				0.134	0.191	0.893
Yes	6 (9.9)	7 (19.4)	6 (18.2)			
No	60(90.1)	29(80.6)	27 (81.8)			
Bone				0.266	0.177	1.000
Yes	13 (19.7)	4 (11.1)	3 (9.1)			
No	53(80.3)	32 (88.9)	30 (90.9)			
Biomarkers						
CPS				0.685	NA	NA
<5	23 (34.8)	14 (38.9)	NA			
≥5	43(65.2)	22 (61.1)	NA			
HER2 positive				0.348	0.234	1.000
Yes	12 (18.2)	4 (11.1)	3 (9.1)			
No	54 (81.8)	32 (88.9)	30 (90.9)			
dMMR				1.000	NA	NA
Yes	4 (6.2)	2(5.6)	NA			
No	62(93.8)	34 (94.4)	NA			
EBER positive				1.000	NA	NA
Yes	4 (6.1)	2 (5.6)	NA			
No	62 (93.9)	34 (94.4)	NA			

I-LM(-), patients without liver metastases treated with immunotherapy; I-LM(+), patients with liver metastases treated with immunotherapy; C-LM(+), patients with liver metastases not treated with immunotherapy; BMI, body mass index; SD, standard deviation; ECOG(PS), eastern cooperative oncology group performance status; CPS, combined positive score; HER2, human epidermal growth factor receptor 2; dMRR, deficient of mismatch repair gene; EBER, Epstein–Barr virus-encoded regions; NA, not applicable.

^1^Calculated as I-LM(-) vs I-LM(+).

^2^Calculated as I-LM(-) vs C-LM(+).

^3^Calculated as P&: I-LM(+) vs C-LM(+).

### Treatment regimen, response, and survival analysis in I-LM(-) group, I-LM(+) group, and C-LM(+) group

As shown in **Table 2**, 43 of 66 (65.2%) patients in the I-LM(-) group and 30 of 36 (83.3%) patients in the I-LM(+) group were treated with immunotherapy plus chemotherapy, 29 of 33 (87.9%) patients were treated with conventional chemotherapy alone in the C-LM(+) group.

**Table 2:** Regime and best treatment response.

	Patients, No. (%)			
Characteristic	All (n=135)	I-LM (-) (n=66)	I-LM +) (n=36)	C-LM (+) (n=33)
Regimen				
Chemotherapy	29 (21.5)	NA	NA	29(87.9)
Chemotherapy plus targeted therapy	4 (3.0)	NA	NA	4 (12.1)
Immunotherapy	11 (8.1)	9 (13.6)	2 (5.6)	NA
Immunotherapy plus chemotherapy	73 (54.1)	43 (65.2)	30 (83.3)	NA
Immunotherapy plus chemotherapy and targeted therapy	18 (13.3)	14 (21.2)	4 (11.1)	NA
Best treatment response				
Complete response	14 (10.4)	9 (13.7)	4 11.1)	1 (3.0)
Partial response	37 (27.4)	22 (33.3)	10 (27.8)	5 (15.2)
Stable disease	31 (23.0)	15 (22.7)	7 (19.4)	9 (27.3)
Progressive disease	53 (39.2)	20 (30.3)	15 (41.7)	18 (54.5)
Objective response rate	51 (37.8)	31 (47.0)	14 (38.9)	6 (18.2)
Disease control rate	82 (60.7)	46 (69.7)	21 (58.3)	15 (45.5)

I-LM(-), patients without liver metastases treated with immunotherapy; I-LM(+), patients with liver metastases treated with immunotherapy; C-LM(+), patients with liver metastases not treated with immunotherapy; NA, not applicable.

All patients were evaluated for the best therapeutic response. Of the 53 patients with progressive disease (PD), 33 had liver metastases, with 20 of 66 (30.3%) patients in the I-LM(-) group, 15 of 36 patients (41.7%) in the I-LM(+) group, and 18 of 33 patients (54.5%) in the C-LM(+) group. The partial response (PR) rate in the I-LM(-) group (33.3%) was higher than that in the I-LM(+) group (27.8%) and the C-LM(+) group (15.2%). The complete response(CR) rate in the I-LM(-) group (13.7%) was also higher than that in the I-LM(+) group (11.1%) and the C-LM(+) group (3.0%). Thus, the objective response rate (ORR) and disease control rate (DCR) in the I-LM(-) group was higher than that in the I-LM(+) group and the C-LM(+) group.

Kaplan-Meier analysis demonstrated a median PFS of 8.7 months in the I-LM(-) group, 4.9 months in the I-LM(+) group, and 4.3 months in the C-LM(+) group(P< 0.001, **Figure 2B**), which showed a median PFS with significant difference between the I-LM(-) group and the I-LM(+) group (P=0.026) but without significant difference between the I-LM(+) group and the C-LM(+) group (P=0.219). The 6-month PFS rates for patients in the I-LM(-) group, I-LM(+) group, and C-LM(+) groups were 65.1% (43 of 66), 41.6% (15 of 36), and 39.3% (13 of 33), respectively. The median OS was 13.43 months in the I-LM(-) group, 10.53 months in the I-LM(+) group, and 8.68 months in the C-LM(+) group (P=0.014, **Figure 2D**), while there was no significant difference between the I-LM(-) group and the I-LM(+) group (P=0.584).

### Analysis of patients with liver metastases treated with immune checkpoint inhibitors

**Figure 3:**
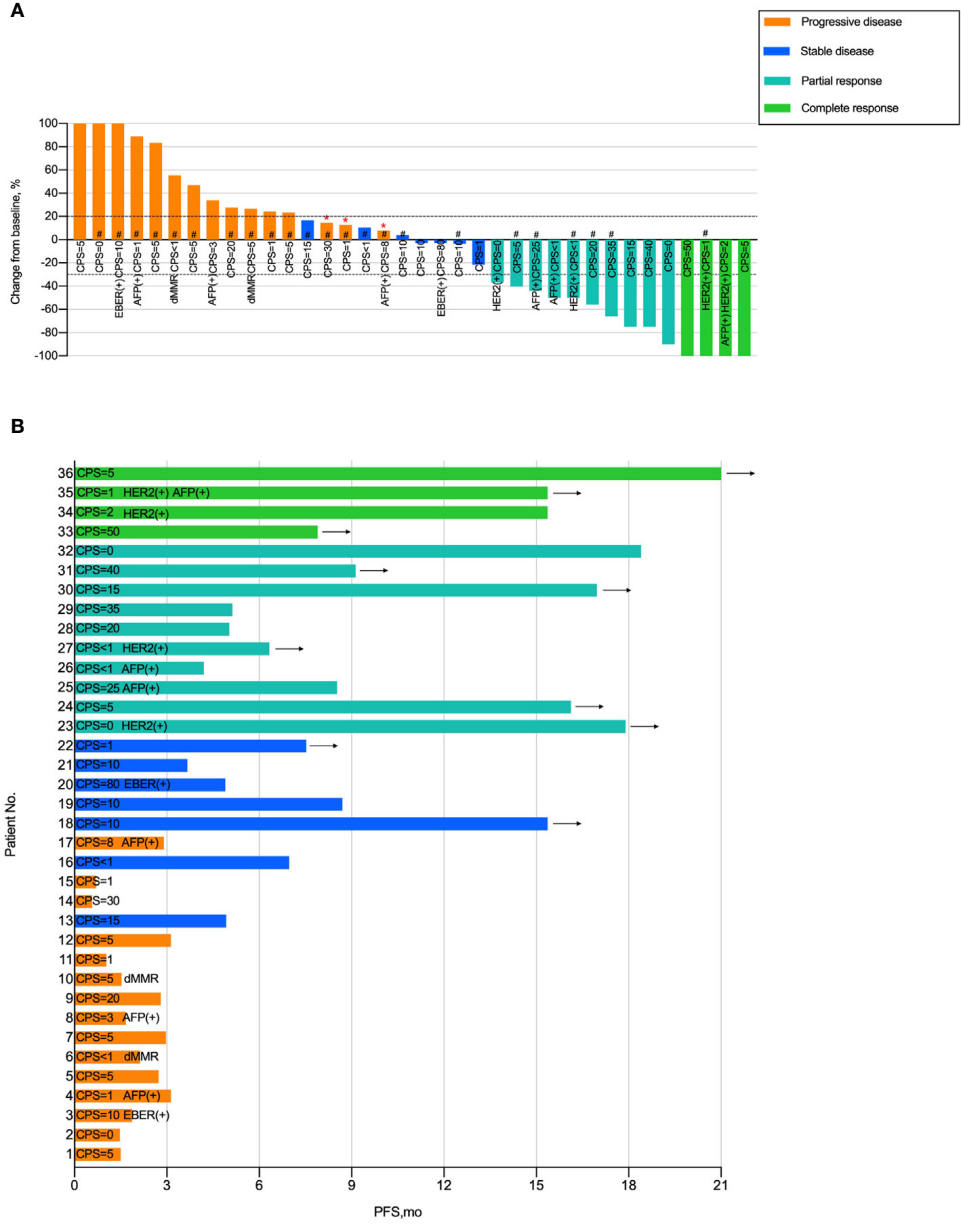
Tumor Response in Patients with Gastric Cancer Liver Metastases Treated with Immunotherapy **(A)** Waterfall plot of tumor response in patients with liver metastases in the I-LM(+) group. **(B)** Swimmer plot presentation of progression-free survival in patients with liver metastases in the I-LM(+) group. CPS, combined positive score; HER2, human epidermal growth factor receptor 2; dMRR, deficient of mismatch repair gene; EBER, Epstein–Barr virus-encoded regions. “*” Indicates new lesion. “#” Indicates presence of peritoneal metastases. Arrow indicates ongoing benefit.

To further demonstrate the clinical benefit of immunotherapy in patients with liver metastases, we performed data analysis of waterfall plots (**Figure 3A**) and swimmer plots (**Figure 3B**) according to the I-LM(+) group. As shown in **Figure 3**, the rate of PD and CR was 41.7% (15 of 36) and 11.1% (4 of 36), respectively (**Figure 3A**). In addition, up to 33% (13 of 36) patients progressed within 3 months and only 22% (8 of 36) patients had a median PFS longer than 12 months. Durable tumor responses (PFS >12 months) were recorded in 10 of 36 (27.7%) patients and the benefit was still ongoing (**Figure 3B**).

To explore the clinicopathologic characteristics associated with PFS in patients with liver metastases treated with immune checkpoint inhibitors, we performed a regression model risk assessment in I-LM(+) group (**Table 3**). Univariate analysis revealed that risk factors associated with PFS included peritoneal metastases (HR, 3.84; 95% CI, 1.42-10.37; P=0.008), metastases sites ≥3 (HR, 2.24; 95% CI, 1.00-5.01; P=0.049) and MMR mutation (HR, 5.33; 95% CI, 1.09-25.9; P=0.038). None of the other variables were associated with PFS outcome, including the status of CPS, HER2, EBER, AFP, and LDH. With multivariate analysis after the adjustment for these significant covariates, only the presence of peritoneal metastases was significantly associated with worse PFS (HR, 3.23; 95% CI, 1.12-9.32; P=0.030). As shown in **Figure 2C**, in the I-LM(+) group, the patients with peritoneal metastases had a significantly shorter median PFS compared with the patients without peritoneal metastases (18.4 months vs 3.1 months, P=0.004); the HER2-negative patients had a worse median PFS compared with the HER2-positive patients (3.95 months vs not reached, P=0.060).

**Table 3:** PFS and association with clinicopathologic characteristics using cox regression in I-LM(+) group.

	Univariate analysis		Multivariate analysis	
Clinicopathologic variable	HR (95% CI)	P value	HR (95% CI)	P value
Age ≥60 vs <60 y	0.70 (0.31-1.58)	0.396	NA	NA
Female vs male	0.49 (0.19-1.24)	0.117	NA	NA
ECOG status 1 vs 0	0.77 (0.10-5.79)	0.798	NA	NA
Tumor size ≥5cm vs <5cm	1.46 (0.66-3.24)	0.344	NA	NA
Differentiation, Poorly VS Well or moderately	1.03 (0.46-2.31)	0.936	NA	NA
Metastases site involved vs noninvolved				
Lymph nodes	1.30 (0.51-3.30)	0.570	NA	NA
Peritoneum	3.84 (1.42-10.37)	0.008	3.23 (1.12-9.32)	0.030
Liver metastases number ≥3	1.03 (0.46-2.31)	0.935	NA	NA
Lung	1.58 (0.62-4.00)	0.333	NA	NA
Bone	0.93 (0.28-3.14)	0.918	NA	NA
Metastases sites ≥3 vs <3	2.24 (1.00-5.01)	0.049	1.37 (0.57-3.31)	0.475
Drug therapy or biomarker involved vs noninvolved				
Targeted therapy	0.18 (0.02-1.34)	0.095	NA	NA
CPS ≥5 vs <5	1.01 (0.45-2.23)	0.975	NA	NA
HER2 positive	0.18 (0.02-1.34)	0.095	NA	NA
MMR mutation	2.31 (1.09-25.9)	0.038	3.29 (0.65-16.68)	0.150
EBER positive	0.26 (0.53-10.1)	0.264	NA	NA
Elevated AFP^1^	1.87 (0.81-4.28)	0.138	NA	NA
Elevated LDH^2^	1.66 (0.75-3.68)	0.211	NA	NA

HR, hazard ratio; Cl, confidence interval; ECOG(PS), eastern cooperative oncology group performance status; CPS, combined positive score; HER2, human epidermal growth factor receptor 2; dMRR, deficient of mismatch repair gene; EBER, Epstein–Barr virus-encoded regions; AFP, alpha-fetoprotein; LDH, lactate dehydrogenase; NA, not applicable.

^1^Elevated AFP represent that Serum AFP concentration ≥25 ng/mL.

^2^Elevated LDH represent that Serum LDH concentration ≥199 ng/mL.

## Discussion

Previous studies revealed that the presence of liver metastases was associated with inferior survival for patients with gastric cancer (20, 21). Many clinical trials have demonstrated promising clinical activity of immunotherapy in advanced gastric cancer, but whether patients with liver metastases could also receive similar benefits is controversial (1–3, 16). Accumulating evidence has revealed that the interaction between tumor cells and the host immune system induced tumor immune evasion, which leads to tumor propagation, recurrence, and metastases (22). One of the mechanisms of immunotherapy was that it could utilize tumor-infiltrating effector CD8^+^ T cells to induce long-lasting tumor treatment responses (23). In the setting of autoimmune illnesses, viral infections, and organ transplantation, the liver with metastatic tumor would promote immunological tolerance and undermine efficient immune synapses, resulting in T cell anergy, regulatory T cell induction, or effector T cell elimination (24, 25). In the CheckMate-649 trial, there were 208 patients in the Chinese subgroup, including 106 patients with liver metastases (26), and the results showed that in all randomized patients, the median PFS in the Nivolumab plus chemotherapy arm was significantly longer than that in the chemotherapy arm (8.3 vs 5.6 months; HR, 0.57; 95% CI, 0.40-0.80), and the ORR was also significantly higher in Nivolumab plus chemotherapy arm (59% vs 41%). Despite the encouraging positive results, the CheckMate-649 trial included a large number of patients with liver metastases, and patients with liver metastases might overshadow the better efficacy of immunotherapy.

The participants in our study were divided into 3 groups based on the presence of liver metastases and immunotherapy, we found a median PFS with significant differences between the I-LM(-) group and the I-LM(+) group (8.7 months vs 4.9 months, P=0.026). In addition, compared with the I-LM(+) group, the ORR was higher(47% vs 38.9%) in the I-LM(-) group. And for all the patients with liver metastases, the patients treated with immunotherapy showed a better response compared with those undergoing chemotherapy and/or targeted therapy (ORR, 38.9% vs 18.2%). As is shown in **Figure 3B**, 15 of 36 (41.6%) patients in the I-LM(+) group had the best tumor response with PD who had a PFS of fewer than four months. In summary, our findings show that individuals with gastric cancer who have progressed with liver metastases may be resistant to immunotherapy. Given inconsistent immunotherapy regimens and the high proportion of peritoneal metastases that were confirmed associated with exactly poor prognostic in previous studies (27), for the patients treated with immunotherapy in our study, the ORR was lower than that in the Nivolumab plus chemotherapy arm in the CheckMate-649 trial (44% vs 58%). Furthermore, considering that immunotherapy has a “survival tailing effect”, the median OS was no significant difference between the I-LM(-) group and the I-LM(+) group (13.43 vs 10.53 months, P=0.584) with a long follow-up. The results indicate that immunotherapy shows promising and long-term antitumor immune response for gastric cancer regardless of liver metastases, which is consistent with REGONIVO trial (28).

Immunotherapy is approved in selected cases of gastric cancer, but the correlation between biomarkers and prognosis is still unclear (29). Several potential predictive biomarkers have been identified in some exceptional responders with gastric cancer, such as PD-L1, HER2, MMR, and EBER (1, 30, 31). To explore whether these immunotherapy-related biomarkers could also predict outcomes for patients with liver metastases of gastric cancer, we analyzed the prognostic risk of these biomarkers and found that compared with the patients with CPS<5, the patients with CPS≥5 had longer median PFS and a lower risk of death (11.5 vs 6.6 months; HR, 0.49; 95%Cl, 0.25-0.94; P=0.032) in the I-LM(-) group, but that was not consistent in the I-LM(+) group (4.9 vs 4.2 months; HR, 1.01; 95%Cl, 0.45-2.23; P=0.97). It may be related to the increasing Treg cells in the tumor microenvironment of the liver metastases, which is now thought to be the “culprit” that induces the tumor cells to evade immune surveillance of the body (32). Kumaga et al. found that the higher levels of glycolysis and lactate in the microenvironment with liver metastases of tumor may lead to the significant reduction of CD8^+^ T-cells + PD-1 expression in tumor tissue, but the increase of Treg cells + PD-1 expression which causes immune tolerance (33). Besides, in the I-LM(+) group, the dMMR was a risk factor for survival, and the best treatment response of the two patients was PD (100%), but with the OS of 12.9 months and 4.3 months, respectively. There are no reports on the correlation between liver metastases and dMMR in gastric cancer, growing evidence have revealed that dMMR is a predictive biomarker for patients with gastric cancer treated with immunotherapy (34). Tumors with dMMR have a high tumor mutational burden, tumor infiltrating lymphocyte enrichment in the microenvironment, and high expression of immunological checkpoint proteins such as PD-1, PD-L1, and CTLA-4 (35). The CheckMate-032 trial subgroup analysis showed that patients with advanced gastric cancer and dMMR had substantial short-term clinical benefits and longer-term survival benefits regardless of the treatment with Nivolumab and/or not Ipilimumab (36). A study analyzing the treatment response of immunotherapy in patients with dMMR showed inconsistent efficacy in several clinical trials [ KEYNOTE-059 (n=7, ORR=57.1%), KEYNOTE-061 (n=15, ORR=46.7%) and KEYNOTE-062 (n=14, ORR=57.1%)] (37). Given the small sample size of patients with dMMR, the predictive value of dMMR in immunotherapy of gastric cancer patients with liver metastases needs further prospective clinical validation. In addition, in the I-LM(+) group, the best treatment response in two EBER-positive patients was PD and SD, respectively. Commonly, the results were variable among previous studies and the predictive efficacy of EBER status for immunotherapy needs prospective studies to validate (38, 39). Furthermore, several previous studies showed that elevated AFP and ADH levels were associated with poor prognosis in some tumors treated with immunotherapy due to the presence of liver metastases (9, 40), but our study found no correlation between these two factors and the prognosis of patients with liver metastases. The inconsistent results might be associated with the strong heterogeneity and the tumor microenvironment of gastric cancer. Previous research had shown that except for hepatic carcinoma, various human tumors (such as gastric cancer, colorectal cancer, gallbladder cancer, lung cancer, and ovarian cancer) could also cause elevated serum AFP, of which gastric cancer was the most prevalent (41). And the tumor microenvironment of different types of tumors with liver metastases may potentially affect the production of LDH. Recent studies have demonstrated that when the tumor with liver metastatic lesions, the high expression level of monocarboxylate transporter 1, high-lactic acid, and hypoxic environments in the internal environment could induce systemic immune tolerance (33). Moreover, unlike later-line immunotherapy in previous studies (10), all immunotherapy regimens in our study were first-line treatments, which might cause inconsistent results.

The peritoneum is considered one of the most common metastatic sites in gastric cancer patients, ranking second after the liver (42, 43). Although several potential biomarkers (such as PD-L1, dMMR, and tumor mutational burden) were demonstrated for gastric cancer patients to predict the superior efficacy of immunotherapy, peritoneal metastases show PD-L1 expression less frequently (44). The immune checkpoint molecule PD-1 and its ligands are unlikely to be possible therapeutic targets for gastric cancer patients with peritoneal metastases, which was confirmed in some previous research (45, 46). In our population, we demonstrate that the presence of peritoneal metastases is an independent risk factor associated with poor prognosis in gastric cancer patients, extremely worse accompanied with liver metastases. In the I-LM(+) group, patients without peritoneal metastases had a superior median PFS of 18.4 months and DCR of 84.6% (11 of 13), compared to 3.1 months and 56.5% (13 of 23) in patients with peritoneal metastases. Besides, for the patients with peritoneal metastases but without liver metastases treated with immunotherapy, the median PFS was 7.0 months and the DCR was up to 63.3% (31 of 49) (**Supplementary Figure 1**). As shown in **Figure 3**, 13 of 15 patients with the best treatment response of PD had peritoneal metastases and had a median PFS shorter than 4 months.

The presence of tumor antigens in the liver metastases could lead to systemic antitumor suppressive immunity and the dysfunctional immune state could not be reversed by anti–PD-1 monotherapy (47). The previous research showed that gastric cancer with liver metastases was associated with higher rates of HER2 positivity (48, 49). After anti-HER2 treatment, for the tumor with HER2 amplification, the release of cytokines such as CCL2, CCL21, VEGF, and CXCL1 was downregulated, and the immunosuppressive factors of the tumor microenvironment were improved (50). Immunotherapy combined with anti-HER2 target therapy may improve the efficacy of immune checkpoint inhibitors. The interim results of the KEYNOTE-811 trial showed that Pembrolizumab plus trastuzumab and chemotherapy could improve ORR to 74.4% in HER2-positive advanced gastric cancer patients (4). In our population, 16 patients with HER2-positive status were treated with immunotherapy plus trastuzumab and chemotherapy, the ORR was up to 81.3% (13 of 16), and the ORR of 4 patients with liver metastases was 100% (2 complete radiographic responses and 2 partial responses). Our study demonstrates that immunotherapy combined with anti-HER2 treatment could significantly improve survival benefits in gastric cancer patients with HER2-positive and liver metastases.

To our knowledge, our study is the first to investigate the clinical response and PFS in a multivariable analysis that includes various biomarkers of PD-1 targeting therapy in a population with liver metastases of gastric cancer. We acknowledge that our study has limitations. Because this is a retrospective cohort study with small sample sizes that has selective deviation. Furthermore, the regimens of immunotherapy, combination targeted therapy, and chemotherapy are not fully aligned, which may affect the outcomes. Even so, this data truly reflects the clinical treatment efficacy and prognosis-related risk factors of gastric cancer patients with liver metastases, hoping to provide some reference value for such patients treated with immunotherapy.

## Conclusion

The results of this cohort study demonstrate that the presence of liver metastases is associated with resistance to immunotherapy in gastric cancer patients. Among patients with liver metastases of gastric cancer, HER2-positive patients may derive clinical benefits from immune checkpoint inhibitors, while the presence of peritoneal metastases is associated with resistance. Additional preclinical and clinical work should be exerted to identify and overcome mechanisms of immunotherapy in gastric cancer with liver metastases.

## Data availability statement

The original contributions presented in the study are included in the article/**Supplementary Material**. Further inquiries can be directed to the corresponding author.

## Ethics statement

The study was reviewed and approved by the Ethics Committee of Nanfang hospital, Southern Medical University and was conducted in accordance with this committee’s regulations and the Declaration of Helsinki. The patients/participants provided their written informed consent to participate in this study.

## Author contributions

Concept and design: HYL, LYZ. Acquisition, analysis, or interpretation of data: HYL, YQ, CW, ZX. Drafting of the manuscript: HYL, ZL, ZW. Critical revision of the manuscript for important intellectual content: GL, HL, LZ. Statistical analysis: HYL, CW, YQ, FL, YL, XC. Obtained funding: LZ. Supervision: LZ. All authors contributed to the article and approved the submitted version.

## Funding

This study was supported by grant 81902444 from the National Natural Science Foundation of China; grant 2020A1515010269 from the Natural Science Foundation of Guangdong Province; grant 201903010072 from the Science and Technology Program of Guangdong Province.

## Conflict of interest

The authors declare that the research was conducted in the absence of any commercial or financial relationships that could be construed as a potential conflict of interest.

## Publisher’s note

All claims expressed in this article are solely those of the authors and do not necessarily represent those of their affiliated organizations, or those of the publisher, the editors and the reviewers. Any product that may be evaluated in this article, or claim that may be made by its manufacturer, is not guaranteed or endorsed by the publisher.
