# Supplementary material for: Prognostic characteristics and clinical response to immunotherapy targeting programmed cell death 1 for patients with advanced gastric cancer with liver metastases
Source: Front Immunol. 2022 Sep 21;13:1015549. doi: 10.3389/fimmu.2022.1015549 (PMC9532548; doi:10.3389/fimmu.2022.1015549)
Supplement: Supplementary file 1 [file DataSheet_1.docx]

**Supplement table**

eTable 1. Baseline characteristics of all patients before first treatment with and without liver metastases.

eTable 2. PFS and association with clinicopathologic characteristics using cox regression in all patients.

| **eTable 1. Baseline characteristics of all patients before first treatment with and without liver metastases** | | | | |
| --- | --- | --- | --- | --- |
|  | **Patients, No. (%)** |  |  |  |
| **Characteristic** | **All participants**  **(n=135)** | **LM(-)**  **(n=66)** | **LM(+)**  **(n=69)** | **P value^b^** |
| Demographic |  |  |  |  |
| Gender |  |  |  | 0.101 |
| Male | 89(65.9) | 39(59.1) | 50(72.5) |  |
| Female | 46(34.1) | 27(40.9) | 19(27.5) |  |
| Age, median (IQR), y | 56(21-76) | 55(20-76) | 56(21-76) | 0.442 |
| Age, y |  |  |  | 0.614 |
| <60 | 83(61.5) | 42(63.6) | 41(59.4) |  |
| ≥60 | 52(38.5) | 24(36.4) | 28(40.6) |  |
| Clinical |  |  |  |  |
| BMI,mean(SD)^a^ | 21.26(3.58) | 21.01(3.37) | 21.49(3.77) | 0.433 |
| ECOG(PS) |  |  |  | 0.103 |
| 0 | 116(85.9) | 60(90.9) | 56(81.2) |  |
| ≥1 | 19(14.1) | 6(9.1) | 13(18.8) |  |
| Primary tumor location |  |  |  | 0.015 |
| Upper | 27(20.0) | 14(22.1) | 13(18.8) |  |
| Middle | 30(22.2) | 18(27.3) | 12(17.4) |  |
| Lower | 56(41.5) | 30(45.5) | 26(37.7) |  |
| Mixed | 22(16.3) | 4(6.1) | 18(26.1) |  |
| Primary tumor size, cm |  |  |  | 0.019 |
| <5 | 65(48.1) | 25(37.9) | 40(58.0) |  |
| ≥5 | 70(51.9) | 41(62.1) | 29(42.0) |  |
| Histological grade | |  |  | 0.103 |
| Well or moderately differentiated | 20(14.8) | 10(15.2) | 10(14.5) |  |
| Poorly differentiated | 104(77.0) | 54(81.8) | 50(72.5) |  |
| Unknown | 11(8.2) | 2(3.0) | 9(13.0) |  |
| Site of distant metastases | |  |  |  |
| Lymph nodes^c^ |  |  |  | 0.317 |
| Yes | 26(19.3) | 15(22.7) | 11(15.9) |  |
| No | 109(80.7) | 51(77.3) | 58(84.1) |  |
| Peritoneum |  |  |  | 0.020 |
| Yes | 87(64.4) | 49(74.2) | 38(55.1) |  |
| No | 48(35.6) | 17(25.8) | 31(44.9) |  |
| Lung |  |  |  | 0.103 |
| Yes | 19(14.1) | 6(9.1) | 13(18.8) |  |
| No | 116(85.9) | 60(90.9) | 56(81.2) |  |
| Bone |  |  |  | 0.118 |
| Yes | 20(14.8) | 13(19.7) | 7(10.1) |  |
| No | 115(85.2) | 53(80.3) | 62(90.9) |  |
| Abbreviations: I-LM(-),patients without liver metastases; LM(+), patients without liver metastases; BMI, body mass index; SD, standard deviation; ECOG(PS), eastern cooperative oncology group performance status.  a Body mass index is calculated as weight in kilograms divided by height in meters squared.  b Calculated using the Chi-square test or the Fisher exact test except for BMI and Age(T-test) between LM(-) group and LM(+) group.  c Lymph node metastases does not include perigastric lymph node metastases.   \| **eTable 2. PFS and association with clinicopathologic characteristics using cox regression in all patients** \| \| \| \| \| \| --- \| --- \| --- \| --- \| --- \| \|  \| **Univariate analysis** \| \| **Multivariate analysis** \| \| \| **Clinicopathologic variable** \| **HR (95% CI)** \| **P value** \| **HR (95% CI)** \| **P value** \| \| Female vs male \| 0.85(0.54-1.31) \| 0.469 \| NA \| NA \| \| ECOG status 1 vs 0 \| 1.22(0.70-2.12) \| 0.477 \| NA \| NA \| \| Tumor size ≥5cmVS<5cm \| 1.03(0.68-1.55) \| 0.871 \| NA \| NA \| \| Differentiation,  Poorly VS Well or moderately \| 1.15(0.65-2.06) \| 0.616 \| NA \| NA \| \| Metastasis site involved vs noninvolved \| \|  \|  \|  \| \| Lymph nodes \| 1.51(0.91-2.49) \| 0.104 \| NA \| NA \| \| Peritoneum \| 2.19(1.38-3.47) \| 0.001 \| 2.42(1.42-4.14) \| 0.001 \| \| Liver \| 2.04(1.34-3.09) \| 0.001 \| 2.07(1.24-3.46) \| 0.005 \| \| Lung \| 1.62(0.95-2.75) \| 0.073 \| NA \| NA \| \| Bone \| 1.87(1.11-3.16) \| 0.018 \| 1.42(0.81-2.50) \| 0.213 \| \| Metastasis sites ≥3 VS <3 \| 2.27(1.49-3.45) \| 0.000 \| 1.69(1.03-2.78) \| 0.038 \| \| Drug therapy involved vs noninvolved \| \|  \|  \|  \| \| Immunotherapy \| 0.49(0.32-0.77) \| 0.002 \| 0.55(0.32-0.94) \| 0.029 \| \| Targeted therapy \| 0.88(0.50-1.53) \| 0.652 \| NA \| NA \| \| Abbreviations: ECOG(PS), eastern cooperative oncology group performance status; HR, hazard ratio; Cl, confidence interval; NA, not applicable. \| \| \| \| \| \|  \| \| \| \| \| | | | | |
