# Supplementary figures and images for: Prognostic characteristics and clinical response to immunotherapy targeting programmed cell death 1 for patients with advanced gastric cancer with liver metastases
Source: Front Immunol. 2022 Sep 21;13:1015549. doi: 10.3389/fimmu.2022.1015549 (PMC9532548; doi:10.3389/fimmu.2022.1015549)

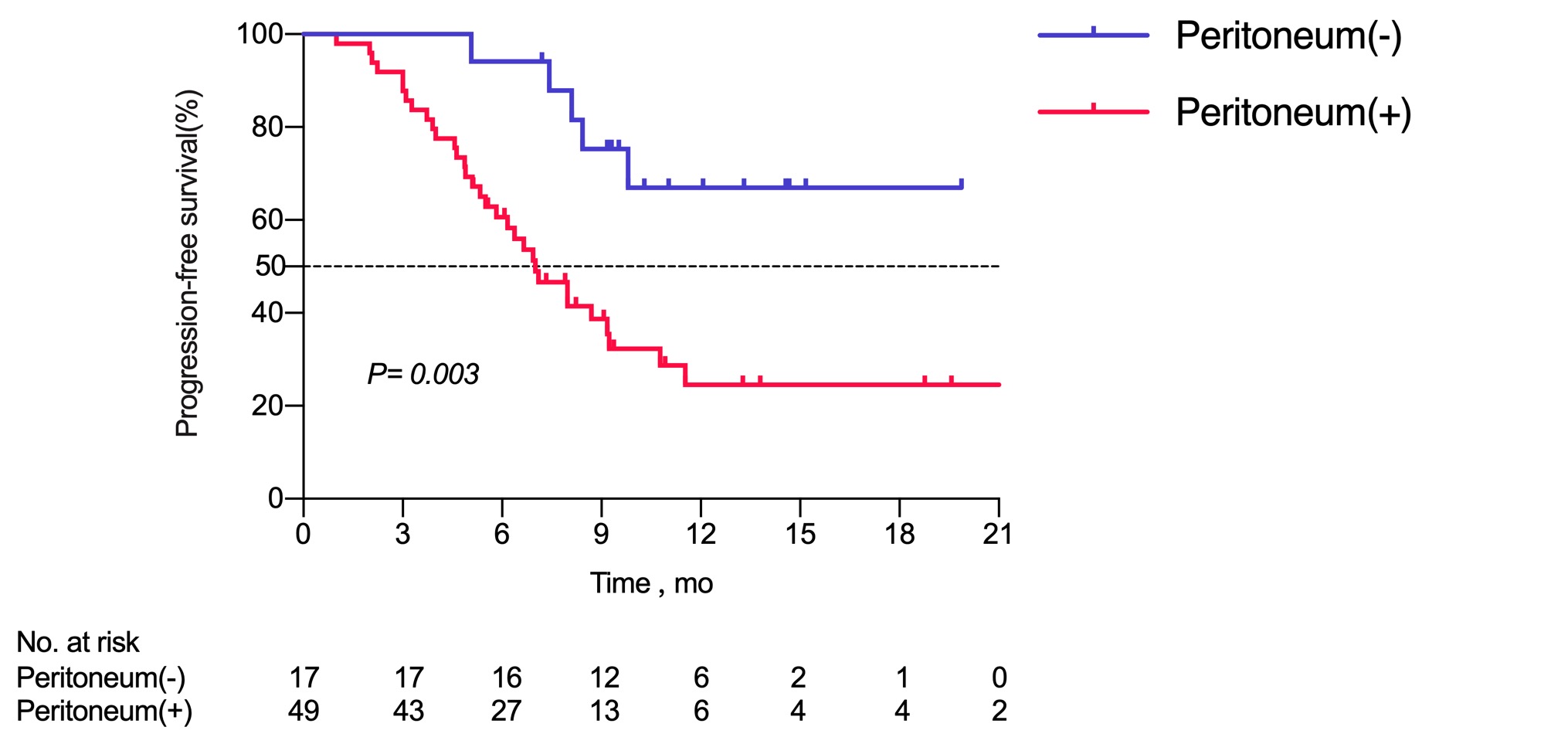

Supplement: Supplementary file 2 [file Image_1.jpeg]
